# The N400/FN400 and Lateralized Readiness Potential Neural Correlates of Valence and Origin of Words’ Affective Connotations in Ambiguous Task Processing

**DOI:** 10.3389/fpsyg.2018.01981

**Published:** 2018-10-30

**Authors:** Kamil K. Imbir, Gabriela Jurkiewicz, Joanna Duda-Goławska, Maciej Pastwa, Jarosław Żygierewicz

**Affiliations:** ^1^Faculty of Psychology, University of Warsaw, Warsaw, Poland; ^2^Biomedical Physics Division, Institute of Experimental Physics, Faculty of Physics, University of Warsaw, Warsaw, Poland

**Keywords:** explicit processing of meaning, ambiguous task, the duality of emotion model, ERP, origin of emotions

## Abstract

Recent behavioral studies revealed an interesting phenomenon concerning the influence of affect on the interpretation of ambiguous stimuli. In a paradigm, where the participants’ task was to read a word, remember its meaning for a while, and then choose one of two pictorial-alphabet-like graphical signs best representing the word sense, we observed that the decisions involving trials with reflective-originated verbal stimuli were performed significantly longer than decisions concerning other stimuli (i.e., automatic-originated). The origin of an affective reaction is a dimension which allows speaking of an affect as automatic (you feel it in your guts) or reflective (you feel it comes from your mind). The automatic affective reaction represents the immediate and inescapable as opposed to the reflective, i.e., the delayed and controllable affective responses to stimuli. In the current experiment, we investigated the neural correlates of performance in an QR-signs-selection ambiguous task. We found the effects of valence and origin in the N400/FN400 potential by means of a stimuli-locked analysis of the initial part of the task, that is, the remembering of a certain word stimulus in a working memory. The N400/FN400 effects were separated in space on scalp distribution. Reflective originated stimuli elicited more negative FN400 than other conditions, which means that such stimuli indeed are associated with conceptual incongruence or higher affective complexity of meaning, but distinct from purely cognitive concreteness. Moreover, the amplitude of the potential preceding the decision, analyzed in the response-locked way, was shaped by the origin of an affective state but not valence. Trials involving decisions concerning reflective-originated words were characterized by a more negative amplitude than trials involving automatic-originated and control word stimuli. This corresponds to the observed pattern of response latencies, where we found that latencies for reflective stimuli were longer than for automatic originated or control ones. Additionally, this study demonstrates that the proposed new ambiguous paradigm is useful in studies concerning the influence of affect on decisions.

## Introduction

The way we process information in situations involving ambiguity may be influenced by many different factors, including the current affective state of an individual (Frijda, 2007) as well as affective connotations of stimuli accompanying the situation (Russell, 2003). In recent behavioral studies (Antosz and Imbir, 2017; Imbir, 2017), a very interesting phenomena was found, namely, considering affective dimensions—instead of valence, the origin of an affective state (Imbir, 2015; Jarymowicz and Imbir, 2015) was the most important factor contributing to the response latencies of decisions made under uncertainty. Origin of an affective state represents the engagement of simplified (automatic) versus elaborated (reflective) criteria of evaluation (Jarymowicz, 2012; Imbir, 2015). Origin was measured with use of heart versus mind dichotomy for affective reactions. High validity and reliability of origin assessments was demonstrated on a large number of word stimuli (Imbir, 2016a). The concept of origin was derived from an emotion duality model (Jarymowicz and Imbir, 2015), the theory highlighting diverse mechanisms leading to formation of an emotion.

### Emotion Duality Model

Presently, one may encounter plenty of theories that elaborate on the topic of the duality of mind (Gawronski and Creighton, 2013). As the idea of duality arises from the division of cognitive processes, some authors place emotions in just one of the processing levels—the faster-thinking mode, known as a heuristic or experiential mind (Epstein, 2003; Kahneman, 2011). Therefore, most of the recent works underline the relations between different emotions and both of the thinking systems (Jarymowicz and Imbir, 2015; Imbir, 2016b). Rules of logic reasoning and verbalization may affect some emotional processes (Strack and Deutsch, 2004), or so-called self-conscious emotions, based on a deliberative approach to a triggering situation, and are strongly related to reflective thinking (Lewis, 1995; Weiner, 2005). The level on which the emotional state is processed, analogically to cognitive-related situations, affects one’s view of the situation, the shape of the behavioral response, and it’s time duration (Kahneman, 2011).

Emotions originating from automatic processing are those that appear to be the answer to criteria deriving from evolution (Damasio, 2010). Such criteria may be placed on a pleasant/unpleasant axis, where everything pleasant generates a positive behavioral response, motivating a human to approach the object, whereas the unpleasant stimuli generates a response that motivates one to avoid the object. Such automatic emotional states are also moderators in the process of fulfilling the needs of organisms’ homeostasis. Deprived needs lead to unpleasant state, which initiates motivation to reach anticipated pleasant state (Berridge, 2004; Berridge et al., 2009). What is worth noting is that emotions of automatic origin do not need language to appear in the human mind as they do not require deliberation in order to change one’s behavior—the emotional states appear immediately, with clear valence and without significant effort (Zajonc, 1980). Emotions of such origin can influence generally experienced mood (Russell, 2003). The reflective system generates more complex emotional states. They may be the previously mentioned self-conscious states as well as other emotions that require psychological representations of objects and their appraisal (Arnold, 1969; Lazarus, 1991; Scherer, 2005). Such representations, called evaluative standards (Reykowski, 1989; Rolls, 2000), are constructs of iconic situations that can be compared with previously experienced circumstances, thus leading the participant to feel a certain emotional state.

Emotions of reflective origin require much more effort, as one has to appraise the current state with the declaratively known ideal one; the process of comparison consumes energy (Kahneman, 2011). There is much less effort needed when the biological criteria of automatic system are used. This leads to a commonly used characteristic of the duality of emotion—the time of processing. As deliberate thinking consumes energy and requires more thought operations, it clearly needs more time for processing. On the other hand, evolutionary-based automatic thinking is faster, as its origin creates an emotion-biological signpost, indicating whether a situation is safe or threatening. It is also worth noting that emotions with origin in the reflective system may be more diverse between humans, as they are based on representations of objects and situations acquired in one’s lifespan. It is obvious that such mental constructs may differ between participants. Originating from evolution, automatic emotions have to work similarly in healthy-working minds. Nevertheless, this difference has most to do with shaping of the emotion, whereas the psychological, autonomic nervous system and behavioral reactions should be alike (Kagan, 2007; Imbir, 2016b).

### Emotion–Cognition Interactions in Ambiguity Processing

**FIGURE 1:**
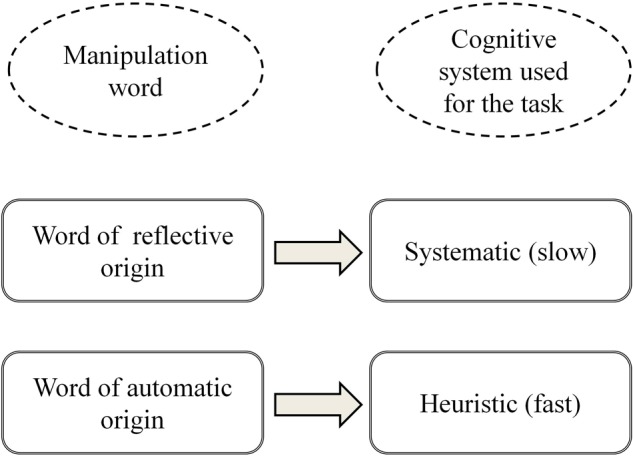
The model of relation between the origin of emotional charge in words and the level of cognitive processing used in the ambiguous task (Imbir, 2016a).

The emotion–cognition model has been proposed on the basis of the above-presented emotion duality postulates (Imbir, 2016b). The model assumes that as both cognition and emotion can be divided into two levels of processing, the interactions between them may occur in four ways within or across systems: (1) emotions of automatic origin influence heuristic thinking (the most recognized way of emotions influencing cognitive processes); (2) reflective emotions affect reflective thinking (e.g., comparing a serious dilemma to one’s moral standards); (3) relations between automatic emotion and reflective thinking (e.g., relationship between generally felt mood and financial decisions; (Lucey and Dowling, 2005); and (4) emotions with origin in the reflective system may influence automatic cognitive processing; returning to moral standards, they may change the influence of the stereotypes (heuristic appraisals) on one’s behavior. Despite the complexity of those within- and cross-system relations, the model assumes that processing emotional stimuli of origin in certain systems biases usage of the same system following cognitive operations. This relation is presented in Figure 1.

In a real life, different situations trigger the usage of one of the two processing systems. Therefore, it is not easy to find a task that does not generically promote one of the thinking levels. It seems that such tasks should have more than one possible correct response (so-called open task), which makes it possible to effectively manipulate mindset or emotional conditions (Raz and Campbell, 2011; Kempe et al., 2013). The crucial element of such a task is the ambiguity of the decision—the state that does not promote any specific processing level, thus making the participant vulnerable to manipulation (Pulford and Colman, 2007). A very famous paradigm that used this type of ambiguous decision was presented by Murphy and Zajonc, 1993. Using ideograms (i.e., symbols from the Chinese language) in this case was accurate, as they were not associated to any specific ideas among people from Western society. This idea, modified to some extent, was also used to create implicit self-reference measures (Błaszczak and Imbir, 2012); Chinese ideograms were substituted with hexagrams, which turned out to be a highly accurate stimuli.

**FIGURE 2:**
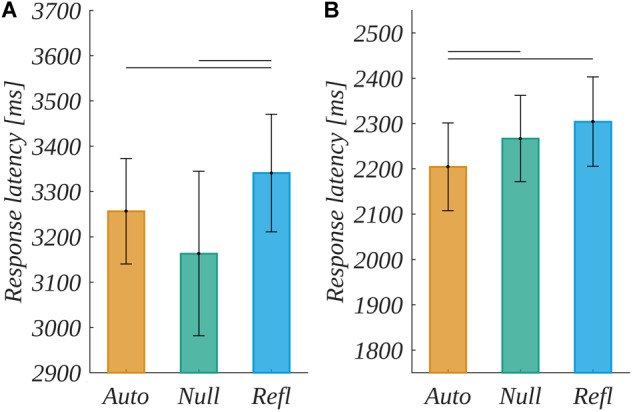
The differences in latencies of deciding in ambiguous task in previous experiments, means for groups in milliseconds for Antosz and Imbir (2017) **(A)**, as well as for Imbir (2017) **(B)**. The bars and SEM are in linear scale, the statistical differences marked by horizontal lines were computed for logarithms of latencies.

The mastered version of the ambiguous task was proposed in recent studies (Antosz and Imbir, 2017; Imbir, 2017). The experiments explored the influence of emotionally charged stimuli on the length of decision latency in conditions of uncertainty. The task for participants was to read and remember a single word, then to identify one of two unknown signs as better representing the meaning of the word stimulus in an unknown far-East sign alphabet. Target stimuli were hexagrams, constructed on a preset pattern: eight horizontal lines divided with randomly placed vertical spaces. The decision latency and, as assumed, level of processing the task differed dependent on origin of the emotion induced by manipulation. In the first experiment (Antosz and Imbir, 2017) using two levels of origin and two levels of valence of word manipulations, the decisions made after presentation of a word with reflective-originated emotional charge took significantly longer than after the automatic-originated stimuli or with no specific system (the control condition was chosen as a baseline, including words that were neutral on both valence and origin). It should be noted, that what differed between the conditions of origin were the response latencies, not the outcome of the decision, which wasn’t ruled by any dependency. Further studies were conducted in a similar paradigm but used a larger set of words as experimental manipulation and confirmed such relation (Imbir, 2017). Reaction latencies for reflective originated stimuli took longer than for automatic originated stimuli. The results for the patterns of reaction times are presented in Figure 2.

What is worth highlighting is that no other properties—neither the experimental stimuli nor the manipulations accompanying the task—accounted for the observed pattern of results caused by origin factor. For example, in both studies, the time spent on reading words was the same for each type of word (automatic vs. reflective). Also, other properties of words, including arousal, concreteness, frequency, and length, were neutral/moderate or aligned across conditions. It is also worth noting that employing a kind of ambiguous decision did not generically promote neither a heuristic nor systematic thinking mode. Whereas the open tasks usually promote deliberative thinking, there is a general tendency to use heuristic thinking due to lower energetic cost and faster processing time (Kahneman, 2011). It seems that equality between both of these attributes was constituted.

### EEG Correlates of Ambiguous Task Processing Components

Taking into account the high consistency of the behavioral pattern of results, we decided to check neural correlates of the task. Considering the ambiguous task as presented in behavioral studies (Antosz and Imbir, 2017; Imbir, 2017), when transmitting it into an EEG paradigm, we must deal with two types of processes: (1) word processing (active, but not requiring immediate answer) with storing a certain stimulus in a working memory; and (2) making a decision concerning certain objects kept in working memory. Each process was investigated earlier with use of ERPs measures; therefore, we briefly present the current state of knowledge in the field of emotional word processing and affect impact on those processes.

ERPs correlates of emotional words were thought to be an interesting method, giving insight into the affect role in word processing (Citron, 2012). So far, different components were identified as indexing cognitive operations during word processing, including early posterior negativity (EPN) (Kissler and Herbert, 2013; Recio et al., 2014) as well as the N400 (Kissler et al., 2009; Scott et al., 2009; Kutas and Federmeier, 2011). Most of the prior knowledge was collected in paradigms requiring active processing in order to create a behavioral response to the certain experimental conditions or trials. The reason for this is the long time required by paradigms measuring ERPs dependent variables (due to a high number of trials), and consequently high probability of losing participants’ attention during only passive reading paradigms. There is one exception: studies involving remembering, working memory, and memory tests afterward (Kissler et al., 2007).

For example, in the study involving passive emotional word processing where it is required to remember the word’s content, early effects were identified for stimuli differing in valence (Kissler et al., 2007). The task for the participant was to try to remember as much as possible from 180 words displayed briefly (333 ms) or longer (1,000 ms). Differences in amplitude were found around 250 ms after stimulus onset (EPN-like component), especially at occipital electrodes. Both positive and negative stimuli elicited more negative amplitude than neutral stimuli. Another instance of neural correlates associated with memory is the N400-like component potential obtained in recollection paradigms. In frontal regions of the brain, the N400 amplitude is thought to be reduced by the familiarity of certain stimuli and enhanced by their relative novelty (Curran, 2000; Mecklinger, 2000). Familiarity impression results from a process of extraction of information from long-term memory but can be contrasted to the effects of working memory load. Working memory is the system that allows us to temporarily store the information in our mind so that we can manipulate this information or act upon it (Smith et al., 1989; Cowan, 2014). The visual working memory load index is the negative slow wave component, a negative deflection succeeding the word processing components (Fukuda et al., 2015). The conceptual and lexical working memory index is the N400 component (Kutas and Federmeier, 2000; Salisbury, 2004), reflecting lexical integration processes. Typically, new information from sensory input is compared to information stored in working memory, and inconsistency elicits a greater negativity in an N400 time range. The sentence processing studies (Wang et al., 2013) showed that earlier parts of the sentence create a context in the mind, and new information is referenced according to this context, providing contextual integration. Such a view emphasizes the importance of the fit between the eliciting item and context-based information. Integration is easier, and the amplitude of the N400 component is correspondingly reduced when the features of a word are coherent with the local context (Kutas and Federmeier, 2000).

The neural correlates of decision-making and movement executing the decision are summarized in the literature as the lateralized readiness potential (LRP) that can be employed as a real-time index of selective response preparation (Kutas and Donchin, 1974; Hackley and Miller, 1995). The LRP exhibits two stages of decision-making: (1) an early signature of gradual evidence accumulation, as well as (2) a later, ballistic, response-related motor component (Van Vugt et al., 2014). The LRP can be observed over the motor cortex contralateral to the responding hand (typically C3–C4 electrodes) and can be observed even before the movement is executed, reflecting the preparation for movement (Smulders and Miller, 2011). The most popular method of LRP scoring is the response-locked averaging across trials (Osman and Moore, 1993; Smulders and Miller, 2011), offering a chance to visualize a peak typically of a few microvolts, which cannot be detected in the ongoing signal.

Considering the usefulness of LRP in cognitive neuroscience, some studies show the susceptibility of this component to task processing specificity (Smulders and Miller, 2011). For example, LRP was found to be a useful measure of response-priming effects (Dehaene et al., 1998). In the experiment, the explicit target task for participants was to perform a simple response selection based on a categorization of the target stimuli (Arabic numbers) with a single rule: if the number is lower than 5, then respond using the left hand; if the number is larger than 5, then respond using the right hand. The task was preceded by a subliminal masked presentation of verbal stimuli (words ascribing numbers), congruently or incongruently priming the information about the hand required to respond to a target task. The LRP showed a significant difference in the predicted direction between congruent and incongruent trials (in incongruent trials, a significant negative deflection indicating motor preparation on the incorrect side of response, and priming information was misleading; whereas in congruent trials there was a non-significant positive deflection). Taking this into account, we may assume that LRP is susceptible to verbal stimuli that offer meaning to the decision-making process. Other studies showed that decisions concerning emotional materials (positive, negative, and neutral pictures from the International Affective Picture System [IAPS] utilized as an emotional stimuli) can result in differences in intervals of LRP. The response-locked LRP interval was shortest for negative pictures, indicating that the negative contents elicited a so-called reaction-priming effect (Huang and Luo, 2006). Taking this into account, we may assume that LRP is susceptible to the affective qualities of stimuli used in a decision task.

### Aims and Predictions

The main aim of the current experiment was to check neural correlates of behavioral phenomena identified in earlier studies (Antosz and Imbir, 2017; Imbir, 2017). The paradigm was adjusted to the requirements of ERPs measurements. In the current study, we also decided to use randomized target stimuli that were as neutral as possible in the form of the QR-codes that were generated from a list of words and represented in a black and white visual pattern. The QR-codes were presented in pairs, the same as in earlier studies; the task for a participant was to choose one of them. We expected on the behavioral level to replicate the pattern of reaction time results, showing larger latencies in ambiguous decisions when reflective originated words were stored in the memory in comparison to automatic originated stimuli. At the decision making and decision execution stage of processing more complex reflective originated stimuli should evoke longer responses due to the fact that the ambiguous task requires careful inspection of two quite similar QR-stimuli and decide which best represent the word stored in memory. The richer evaluative criteria associated with a stimulus (conceptual richness or semantic incongruence), the easier to trigger the multidimensional comparison of QR-code stimulus visual features while performing the decision, and as a result, the longer response latencies should be expected (Antosz and Imbir, 2017; Imbir, 2017).

At the electrophysiological level, we expected to find amplitude differences caused by origin and valence of words in two stages of ambiguous task processing: (1) word reading and remembering the certain stimulus (including stimulus decoding, semantic integration and storing in mind); and (2) at the decision-making stage (including decision preparation and execution).

Considering word reading and remembering, we expected to find some activity associated with the remembering of stimuli, namely, N400/FN400 modulation caused by valence and origin of affective connotations of words and representing semantic integration of stimuli. We expected that the more difficult the trial would be (i.e., word to remember associated with a richer contextual information, like multidimensional criteria of evaluation characteristic to reflectively originated emotions), the more polarized would be the amplitude of ERP’s component. Taking into account earlier results of studies concerning emotional word processing and origin factor (Imbir et al., 2016, 2017), we expected dissociative effects of valence and origin. A valence effect should be present at posterior sites (Kissler et al., 2007), and both positive and negative stimuli should evoke an amplitude different from the amplitude evoked by neutral stimuli. Origin effects were expected at frontal sites (Imbir et al., 2016), because reflective origin represent a stimuli triggering many different associations, including verbalized criteria of evaluation. More complex, reflective-originated verbal stimuli were expected to evoke more effortful processing, therefore higher amplitudes of ERPs in comparison to automatic originated stimuli.

Taking into account decision-making processes, we expected to find the pattern of results resembling the behavioral pattern of reaction latencies. Decisions concerning reflective-originated words should evoke more polarized amplitudes of evoked potential than other conditions. The longer decision latencies index the higher requirements of a task. Decision-related effect should be lateralized in a time window directly preceding the decision (at the decision execution stage of processing), namely, it should be present at the C3 site, but not at the C4 site (responding with the right hand). We also expected that time ranges preceding the final decision execution (at decision preparation stages of processing) would also be affected by the origin factor. Due to the exploratory stage of this experiment, we had no more specific expectations. We applied an exploratory analytic approach in order to investigate the influence of word type on processing the decision. The exploratory approach was based on an assumption, that all time ranges of ERPs and all localizations should be tested in order not to miss any important results not described earlier. The reason for that was only a little prior knowledge about localizations expected to be sensitive to the task.

## Materials and Methods

### Participants

A group of 36 volunteers (18 male and 18 female) aged 19–25 years (*M* = 21.8, *SD* = 1.6) took part in the experiment. They were all students from different Warsaw colleges and universities, native Polish speakers, with normal or corrected to normal vision, right-handed (which was verified in a self made questionnaire before the main experiment). The participants received a small reward for participation in the experiment. Data for two participants were excluded from further analysis due to technical problems, which results in a final group of 34 participants (16 male and 18 female) aged 19–25 years (*M* = 21.8, *SD* = 1.6).

The design, experimental conditions, and consent procedure for this study were approved by the appropriate bioethical committee at Maria Grzegorzewska University. All of the procedures involving human participants were performed in accordance with the ethical standards of the institutional and/or national research committee and with the 1964 Helsinki declaration and its later amendments or comparable ethical standards. Participants provided their verbal informed consent to participate in the presence of at least two lab members, which was documented in a research diary. We did not collect any personal data from our participants, to assure their anonymity.

### Design

The experiment was designed in a 3 (valence) × 3 (origin) model. Both of these independent variables were within-subject factors. The stimuli words were divided into levels of valence: (1) negative, (2) neutral (control), and (3) positive; the origin division was designed analogically: (1) automatic, (2) neutral (control), and (3) reflective. Nine groups of words with 15 words in each group were chosen, each described on both of the experimental dimensions and averaged on others (for a total of 135 words). The target stimuli were random QR-codes presented in pairs. The instructions presented to the subjects were: “try to choose which QR-code better reflects the meaning of the word you will see.” Participants were also told that there is a correct answer, i.e., each pair consist of code generated from the previously displayed word and some other word. The QR-codes, although they actually represented words, were fully randomly assigned to the trials. The participants were not aware of this fact. Subjects were also asked to give the response as soon as possible. The stimuli of interest were words (analyzed in a stimulus-locked way in ERPs) and QR-codes (analyzed in a behavioral manner as well as in a response-locked way in ERPs). The dependent variables were (a) the decision taken in the ambiguous task (selected QR-code, upper or lower); (b) time of making the decision; (c) amplitude of potential evoked during reading the word; and (d) amplitude of potential evoked during deciding in the task.

### Linguistic Materials

The used words were taken from the Affective Norms for Polish Words Reload database (Imbir, 2016a). In this database, affective reactions to words have been measured on nine scales (including valence, origin, arousal, and concreteness). Each word was measured by at least 50 participants and proportionately between men and women. Special Likert scale for origin measurement was prepared for that study, with images attached to different values. The images related to extreme values showed different ends of each dimension, e.g., a simple image of human with giant heart-like shape on chest depicted ‘automatic’ end of the origin scale, while the same silhouette with giant brain depicted ‘reflective’ end. This scale allowed us to measure a general perception of an origin toward a certain stimulus among a student’s population in Poland. We assumed that some stimuli (words) may be understood in a very similar way by a group of people living in similar environmental conditions, despite the fact that affective connotations of stimuli (including valence or arousal) are the matter of a subjective interpretation. On the basis of normative ratings, the means for each word on each of the nine scales were calculated. To prepare stimuli for this experiment, we picked words with boundary values on valence (negative vs. positive) and origin (automatic vs. reflective) scales. We chose four different dimensions that should be controlled among those words: arousal, concreteness, number of letters, and frequency of usage in the Polish language (Kazojć, 2011), transformed into natural logarithms for calculation. Words of no specific valence and origin (control groups) were also chosen. Experimental stimuli constituted nine groups of 15 words each, yielding 135 words in total.

An ANOVA analysis, 3 (valence) × 3 (origin) model, was conducted for all of the dimensions, manipulated and controlled. In the case of valence, the groups differed significantly on the valence dimension, *F*(2,126) = 607.44, *p* < 0.001, η^2^ = 0.91, while no significant differences were observed between ratings on other scales. Analogically in the case of origin there was a significant difference on the origin scale, *F*(2,126) = 1.27, *p* = 0.28, η^2^ = 0.02. There was also a significant effect for number of letters in the case of origin, *F*(4,126) = 0.82, *p* = 0.52, η^2^ = 0.025. Verification by simple contrast analysis showed differences in length between words of automatic origin (*M* = 7.4; *SD* = 2.3) and no particular origin (*M* = 6.2; *SD* = 1.9), *t*(132) = 2.62, *p* = 0.01. No other effects in the case of origin were identified, nor in the case of interaction between dimensions. Descriptive statistics of groups may be found in Table 1; an entire analysis of differences between them is shown in Table 2. The full list of stimuli is available in Table 3 and Appendix Table A1.

**Table 1:** Means and standard deviations for nine groups of words used as the dependent variable.

Level of origin		Level of valence							
	
		Negative		Neutral		Positive		Total	
		*M*	(*SD*)	*M*	(*SD*)	*M*	(*SD*)	M	(*SD*)
Automatic	Valence	3.50	(0.36)	5.02	(0.56)	6.71	(0.35)	5.07	(1.39)
	Origin	4.45	(0.53)	4.58	(0.37)	4.33	(0.70)	4.45	(0.55)
	Arousal	4.37	(0.49)	4.15	(0.55)	4.28	(0.80)	4.27	(0.62)
	Concreteness	4.31	(1.15)	3.95	(0.74)	4.48	(1.20)	4.24	(1.05)
	NoL	7.20	(2.65)	7.47	(1.96)	7.40	(2.41)	7.36	(2.31)
	Ln (freq)	5.21	(1.91)	5.65	(2.03)	5.73	(2.28)	5.53	(2.04)
Control (0)	Valence	3.37	(0.36)	5.19	(0.54)	6.38	(0.32)	4.98	(1.32)
	Origin	5.41	(0.31)	5.49	(0.30)	5.36	(0.35)	5.42	(0.32)
	Arousal	4.15	(0.23)	4.12	(0.67)	4.04	(0.51)	4.11	(0.49)
	Concreteness	4.05	(1.12)	3.96	(1.32)	4.17	(0.74)	4.06	(1.06)
	NoL	6.47	(2.03)	5.27	(1.33)	6.93	(2.02)	6.22	(1.92)
	Ln (freq)	5.48	(2.28)	5.97	(1.27)	6.61	(2.02)	6.02	(1.92)
Reflective	Valence	3.66	(0.35)	5.30	(0.39)	6.49	(0.40)	5.15	(1.23)
	Origin	6.46	(0.30)	6.63	(0.41)	6.63	(0.56)	6.57	(0.43)
	Arousal	4.32	(0.49)	3.93	(0.47)	4.03	(0.36)	4.10	(0.46)
	Concreteness	4.17	(1.13)	4.09	(1.17)	4.41	(1.07)	4.22	(1.11)
	NoL	7.07	(1.75)	6.27	(1.62)	7.20	(2.27)	6.84	(1.91)
	Ln (freq)	5.42	(1.37)	6.53	(1.79)	6.01	(1.22)	5.99	(1.52)
Total	Valence	3.51	(0.37)	5.17	(0.50)	6.53	(0.38)	5.07	(1.31)
	Origin	5.44	(0.92)	5.57	(0.92)	5.44	(1.09)	5.48	(0.97)
	Arousal	4.28	(0.42)	4.07	(0.56)	4.12	(0.58)	4.16	(0.53)
	Concreteness	4.18	(1.11)	4.00	(1.08)	4.35	(1.01)	4.18	(1.07)
	NoL	6.91	(2.15)	6.33	(1.86)	7.18	(2.20)	6.81	(2.09)
	Ln (freq)	5.37	(1.85)	6.05	(1.72)	6.12	(1.89)	5.85	(1.84)

**Table 2:** Differences between groups of words (valence and origin).

	Valence, main effect for groups of words	Origin, main effect for groups of words	Valence and origin interaction
Valence	*F*(2,126) = 607.44, *p* < 0.001, η^2^ = 0.91	*F*(2,126) = 1.88, *p* = 0.16, η^2^ = 0.03	*F*(4,126) = 2.09, *p* = 0.086, η^2^ = 0.062
Origin	*F*(2,126) = 1.27, *p* = 0.28, η^2^ = 0.02	*F*(2,126) = 254.55, *p* < 0.001, η^2^ = 0.80	*F*(4,126) = 0.5, *p* = 0.74, η^2^ = 0.016
Arousal	*F*(2,126) = 1.98, *p* = 0.14, η^2^ = 0.02	*F*(2,126) = 1.44, *p* = 0.24, η^2^ = 0.02	*F*(4,126) = 0.5, *p* = 0.72, η^2^ = 0.016
Concreteness	*F*(2,126) = 1.19, *p* = 0.31, η^2^ = 0.02	*F*(2,126) = 0.4, *p* = 0.67, η^2^ = 0.006	*F*(4,126) = 0.12, *p* = 0.98, η^2^ = 0.004
Number of letters	*F*(2,126) = 2.01, *p* = 0.14, η^2^ = 0.03	*F*(2,126) = 3.48, *p* = 0.034, η^2^ = 0.052	*F*(4,126) = 0.82, *p* = 0.52, η^2^ = 0.025
Frequency	*F*(2,126) = 2.3, *p* = 0.11, η^2^ = 0.04	*F*(2,126) = 1.0, *p* = 0.37, η^2^ = 0.016	*F*(4,126) = 0.44, *p* = 0.78, η^2^ = 0.014

*Statistically significant differences are printed in black.*

**Table 3:** The full list of words stimuli used in each condition.

		Valence category					
		Negative		Neutral		Positive	
**Origin category**	**Automatic**	*Czkawka*	Hiccup	*Procesja*	Procession	*Zakochanie*	Infatuation
		*Szloch*	Sob	*Kościół*	Church	*Passa*	Streak
		*łzy*	Tears	*Kuksaniec*	Nudge	*Toast*	Toast
		*Uszczypni ecie*	Pinch	*Tarot*	Tarot	*Powitanie*	Welcome
		*Pijak*	Drunk	*Loteria*	Lottery	*Zapach*	Fragrance
		*Naiwniak*	Sucker	*Westchnienie*	Sigh	*Słodycz*	Sweetness
		*Słabeusz*	Weakling	*Jałmużna*	Alms	*Pomoc*	Help
		*Zm eczenie*	Fatigue	*Błazen*	Clown	*Niemowlak*	Infant
		*Hałas*	Noise	*Mrowienie*	Tingling	*Flirt*	Flirt
		*Plotka*	Rumor	*Pragnienie*	Desire	*Potomstwo*	Offspring
		*Grymas*	Grimace	*Obrz ed*	Rite	*Pozdrowienie*	
		*Gafa*	Blunder	*Wróżka*	Fairy	*Skarb*	Greeting
		*Usidlenie*	Ensnaring	*Młodzież*	Youth	*Walentynka*	Treasure
		*Smarkacz*	Stripling	*łasuch*	Gourmand	*Podarunek*	Valentine
		*Zaślepienie*	Infatuation	*Burza*	Storm	*Ferie*	Gift Holiday
	**Control (0)**	*Wina*	Fault	*Doping*	Cheering	*Przyj ecie*	Party
		*Ciemnota*	Unacquaintance	*Chór*	Choir	*Rejs*	Cruise
		*Truchło*	Carcass	*Kł ebek*	Hank	*Powiew*	Waft
		*Dół*	Pit	*Telewizja*	Television	*Promocja*	Promotion
		*Ochłap*	Offal	*Guru*	Guru	*Klimat*	Climate
		*Breja*	Slush	*Wódka*	Vodka	*Gość*	Guest
		*Paszkwil*	Libel	*Unik*	Dodge	*Brawa*	Applause
		*Kuternoga*	Lame	*Czara*	Goblet	*Kreskówka*	Cartoon
		*Reumatyzm*	Rheumatism	*Smok*	Dragon	*Melodia*	Melody
		*Biedak*	Wretch	*Blef*	Bluff	*Wydarzenie*	Event
		*Śpi azka*	Coma	*żargon*	Jargon	*Smak*	Taste
		*Obtarcie*	Sore	*Gł ebia*	Depth	*Południe*	South
		*Bł ad*	Error	*Farsa*	Farce	*Malarstwo*	Painting
		*łachmany*	Rags	*Grono*	Bunch	*Wyzwanie*	Challenge
		*Wada*	Drawback	*Pisarz*	Writer	*Obrońca*	Defender
	**Reflective**	*Egzaminy*	Exams	*Szlachta*	Nobility	*Miliard*	Billion
		*Ignorancja*	Ignorance	*Etykieta*	Label	*Tolerancja*	Tolerance
		*Krata*	Grating	*Sułtan*	Sultan	*Mistrz*	Master
		*Minus*	Minus	*Zadatki*	Makings	*Patent*	Patent
		*Szpieg*	Spy	*Prawo*	Right	*Dobytek*	Property
		*Koszty*	Costs	*Prasa*	Press	*Absolwent*	Graduate
		*Podwładny*	Subordinate	*Stawka*	Bid	*Uczony*	Scholar
		*Podatek*	Tax	*Raport*	Report	*Stypendium*	Scholarship
		*Alimenty*	Alimony	*Wojsko*	Army	*Szczyt*	Peak
		*Odsetki*	Interest	*Interes*	Business	*Równowaga*	Balance
		*Rz ad*	Government	*Dyscyplina*	Discipline	*Oszcz edności*	Savings
		*Przemyt*	Smuggling	*Wynik*	Result	*Płaca*	Wages
		*Recesja*	Recession	*Weto*	Veto	*Satyra*	Satire
		*Bezrobocie*	Unemployment	*Hodowla*	Breeding	*Lider*	Leader
		*Heretyk*	Heretic	*Kurs*	Course	*Zysk*	Profit

### Procedure

**FIGURE 3:**
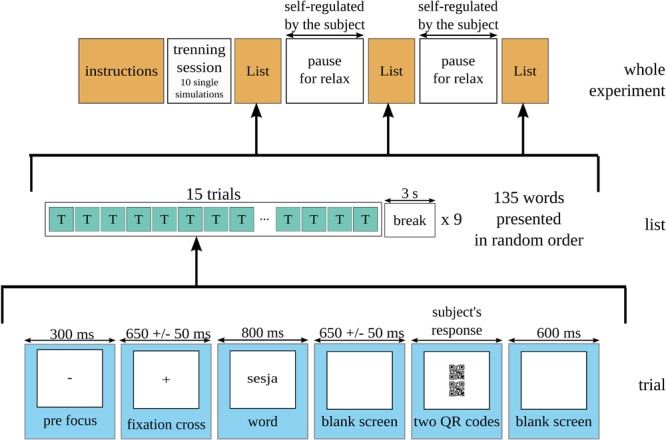
Outline of the experimental procedure. The task for participants was to select the QR-code best representing the word displayed at the beginning of a trial.

The outline of the experimental procedure is depicted in Figure 3. The experimental session consisted of four parts. First, there was a series of 10 rehearsal trials, which were meant to adapt the participant to the experimental conditions. Next, the list of words was presented three times. The list consisted of 135 words selected as stimuli, 15 in each of nine classes obtained as a result of the manipulation of levels of the investigated factors. After every 15 trials, there was a 3-s pause in order to minimize eye fatigue. After each presentation of a full list, there was a self-regulated pause for relaxation. The list of words was presented each time in a random order. Also, for each of the presentations, a pair of QR-codes was randomly assigned to each of the words.

The timing of a single trial was as follows. For the initial 300 ms, there was the “-” sign displayed as a prefocus. Then, a focus sign “+” was displayed for a period of 650 ±50 ms. Next, a stimulus word appeared for 800 ms. From our previous behavioral studies (Antosz and Imbir, 2017; Imbir, 2017) it followed that 800 ms was an average time for a reaction to a word reading task. The stimulus presentation period finished automatically, as we did not want to introduce any movement-related effects or artifacts. After this, the screen went blank for a period of 650 ± 50 ms. Next, the image showing two QR-codes appeared on the screen and stayed on until the participant pressed a response key (up or down arrow on the keyboard). The response was always given with the index or middle finger of the right hand. The trial ended with the blank screen displayed for 600 ms.

### EEG Recording and Analysis

#### Apparatus

A standard personal computer monitor (LCD display; 15-inch diagonal) was used to display stimuli. Monitoring and recording of EEG data were executed with a second personal computer. EEG data were synchronized to the stimuli by means of a custom-made hardware trigger. EEG activity was recorded from 19 electrode sites: Fz, Cz, Pz, Fp1/2, F7/8, F3/4, T7/8, C3/4, P7/8, P3/4, O1/2, referenced to linked earlobes. The ground electrode was placed on the clavicle. The impedance of the electrodes was 5 kΩ or less. Additionally, vertical EOG was recorded from an electrode above the left eye referenced to linked ears. We used Ag/AgCl electrodes. The signal was acquired using a Porti7 (TMSI) amplifier, with a sampling frequency of 256 Hz. Software “SVAROG^1^” was used for the EEG data monitoring and recording.

#### EEG Offline Processing

The offline processing of the EEG signal was performed in Matlab^®^ with the EEGLAB toolbox (Delorme and Makeig, 2004) and self-made scripts. The signal was zero-phase low- and high-pass filtered. Low-pass filtering was done with use of a Butterworth second-order filter (corresponding to 12 dB/octave roll-off) with half amplitude cut-off frequency 30 Hz. High-pass filtering was done with use of a Butterworth second-order filter (corresponding to 12 dB/octave roll-off) with half amplitude cut-off frequency 0.1 Hz. Additional processing steps were specific to the two types of analysis performed, that is, analysis related to the word reading ERP and to the decision related ERP. These are described in the following sections.

#### Processing of Data Related to Word-Reading

The data was divided into fragments from -200 ms to 850 ms (0 ms is the moment of word appearance). The epochs were baseline-corrected, with baseline taken from -200 ms to 0 ms. From the group of 34 participants, seven were excluded due to more than 50% of trials rejected in any of the valence or origin levels. The rejection of trials was based on contamination with EEG artifacts and was done automatically (rejection of trials with amplitude exceeding ±60 μV and abnormal trends of 50 μV per epoch). Effectively, this part of the analysis proceeded on the data of 27 individuals (12 male and 15 female) aged 19–25 years (*M* = 21.9, *SD* = 1.6).

After the rejection of trials containing EEG artifacts, the mean number of trials for each condition was 35.7 (*SEM* = 0.4). We investigated, with use of a Friedman test for replicated block designs, whether the mean number of trials per condition was not different for the valence groups with origin as a blocking variable [χ^2^(2) = 1.269, *p* = 0.53] and for the origin groups with valence as a blocking variable [χ^2^(2) = 0.242, *p* = 0.87].

**FIGURE 4:**
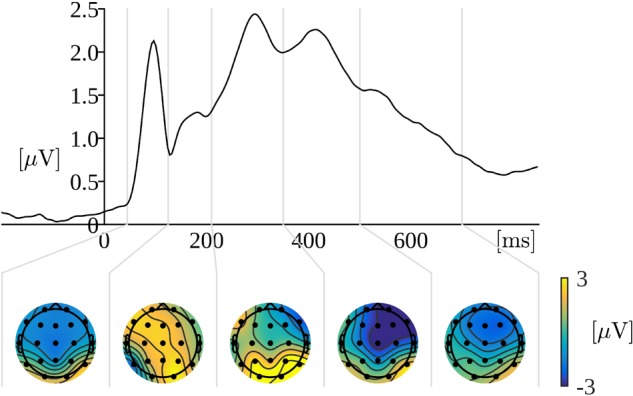
Upper curve: Global field power for the word reading part of the trials. Time 0 is the onset of a word display. Lower plots: Topographical distribution of grand mean amplitudes across participants and conditions within the indicated time windows.

Based on the timing of consecutive maxima in the global field power (GFP) curve (Figure 4) the following time ranges for analysis of the amplitude were selected: 45–125 ms, 125–210 ms, 210–350 ms, 350–500 ms, and 500–700 ms. The GFP was evaluated as spatial standard deviation. It quantifies the sum of electrical activity over all electrodes at a given time point. The latencies of GFP maxima may be interpreted as the latencies of evoked potential components (Lehmann and Skrandies, 1980; Skrandies, 1990).

Based on topographical distributions of amplitude (averaged across the corresponding time window, conditions, and participants) depicted in the Figure 4, we selected four regions of interest (ROI): left-frontal (LF: Fp1, F3, F7), right-frontal (RF: Fp2, F4, F8), left-parietal (LP: P3, P7, O1), and right-parietal (RP: P4, P8, O2). This choice of ROIs allows to take exploratory approach in which we can examine both contrasts: frontal versus parietal and left versus right.

#### Processing of Data Related to Decision

Data related to the decision, due to the nature of the task (comparison of upper and lower QR-code) were contaminated by ocular artifacts. To clean the data from the saccades, the blink artifacts, and from electrical line noise (50 Hz) we used the independent component analysis (ICA) (Makeig et al., 1996). We utilized the Infomax ICA algorithm (Bell and Sejnowski, 1995) implemented in the EEGLAB toolbox (Delorme and Makeig, 2004). The algorithm transformed the continuous, filtered signal into 19 temporally independent components. Components related to the brain activity were differentiated from components containing artifacts related to blinks, eye movements and electrical line noise based on visual inspection of time courses, components’ scalp-map, and activity power spectrum. Only the brain-related components were back-projected to the EEG-signal domain by the inverse transform yielding a signal with significantly reduced artifacts.

Trials, in which the decision was shorter than the 5 percentile or longer than the 95 percentile, were excluded from the analysis. Effectively, we analyzed the trials with the latency of the reaction within the limits from 738 to 6,601 ms. The epochs from -800 to 0 ms preceding the response were extracted from the continuous data. The additional automatic artifact rejection was applied to the epoched signal, removing epochs with trends bigger than 50 uV/epoch and abnormal signal values exceeding ±60 uV. After the data rejections due to extreme reaction times or the extremities in the time course, we had to exclude three participants from the group of 34 participants described in the Section “Participants” because they had more than 50% of trials rejected. Effectively, this part of the analysis proceeded on the data of 31 individuals (15 male and 16 female) aged 19–25 years (*M* = 22.0, *SD* = 1.6).

For these participants, the mean number of clean trials for each condition was 38.4 (*SEM* = 0.3). We investigated, with use of a Friedman test for replicated block designs, whether the mean number of trials per condition was not different for the valence groups with origin as a blocking variable [χ^2^(2) = 3.110, *p* = 0.21] and for the origin groups with valence as a blocking variable [χ^2^(2) = 0.581, *p* = 0.75].

**FIGURE 5:**
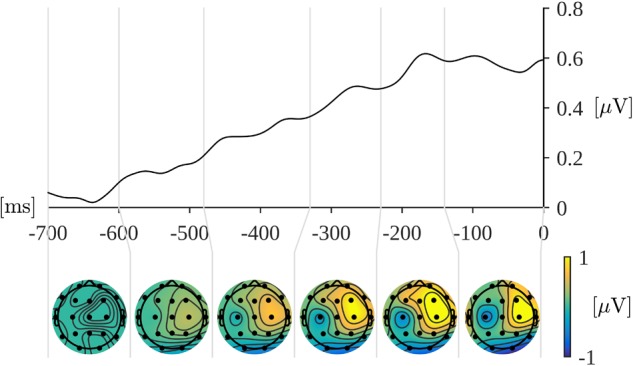
Upper curve: Global field power for the decision-making part of the trials. Time 0 is the moment of key-press. Lower plots: Topographical distribution of grand mean amplitudes across participants and conditions within the indicated time windows.

When we analyzed ERP data aligned with respect to a key-press, there is no *a priori* good baseline period for baseline correction. To overcome the problem, we analyzed the average time course of ERP for each condition without baseline correction (Appendix Figures A1, A2), and found that in the period from -700 to -600 ms the traces for different levels of the analyzed factors are the most similar. Therefore, we used this time period as a baseline for correction of epochs before further analysis. The following time ranges for analysis of the amplitude were chosen: -480 to -330 ms, -330 to -230 ms, -230 to -140 ms, and -140 to 0 ms on the evidence from the timing of consecutive maxima in the GFP curve (Figure 5). We selected four regions of interest (ROI): Fz, Pz, C3, and C4, as we wanted to study the frontal, parietal, and movement-related activity of the brain.

## Results

### Behavioral Results

The analysis of behavioral data was conducted on the same group of participants and trials as in the decision-related ERP analysis (see the Section “Processing of Data Related to Decision”) in order to assure the comparability of results.

#### The Type of QR-Related Decision

With 3 × 3 (valence × origin) repeated measures ANOVA we analyzed the percentage of the number of trials in which participants decided that the upper QR-code represents the earlier displayed word. There was a statistically significant effect of valence, *F*(2,60) = 5.64, *p* < 0.01, reflected in a lower percentage of “up” responses in Neu (*M* = 47.99, *SEM* = 1.25) than in Neg [*M* = 51.17, *SEM* = 1.12; *t*(30) = -3.68, *p* < 0.005] and in Pos [*M* = 50.96, *SEM* = 1.11; *t*(30) = 2.52; *p* < 0.05].

#### Response Latencies

**FIGURE 6:**
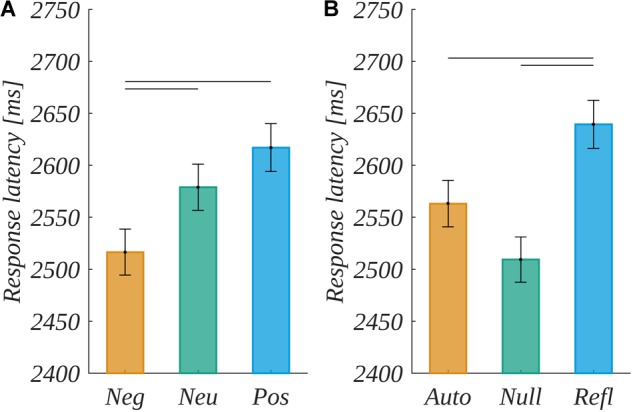
Response latencies for valence **(A)**, and origin **(B)** levels. The bars and SEM are in linear scale, the statistical differences marked by horizontal lines were computed for logarithms of latencies.

Response latencies were measured as the time interval between the moment of QR-code display and the moment of pressing on the response key. Distribution of response latencies is right-skewed and to transform it into approximately normal one, the natural logarithm transformation was used (Heathcote et al., 1991). The normal logarithm-transformed response latencies were analyzed with 3 × 3 (valence × origin) repeated measures ANOVA. A statistically significant effect of valence, *F*(2,60) = 6.335, *p* < 0.01, was found. The response latencies were shorter in Neg (*M* = 2517, *SEM* = 112) than in Neu [*M* = 2579, *SEM* = 114; *t*(30) = 2.52, *p* < 0.05] and Pos [*M* = 2617, *SEM* = 123; *t*(30) = 3.29, *p* < 0.01]. The pattern of changes in mean response latencies is depicted in Figure 6A. There was also a statistically significant effect of origin, *F*(2,60) = 8.105, *p* < 0.001. The response latencies in Refl (*M* = 2639, *SEM* = 121) were longer than in Auto [*M* = 2563, *SEM* = 119.4; *t*(30) = 2.68; *p* < 0.05] and Null [*M* = 2510, *SEM* = 110; *t*(30) = 3.6, *p* < 0.01]. The pattern of changes in mean response latencies is depicted in Figure 6B.

### Effects in Word Reading ERP

#### Time Windows From 45 to 210 ms

The first 210 ms did not yield significant ERP modulations.

#### Time Window From 210 to 350 ms

The interaction between levels of valence, origin, and ROIs, *F*(5.16,134.34) = 3.211, *p* < 0.01, was found statistically significant. Mauchly’s test revealed that the assumption of sphericity had been violated [χ^2^(12) = 0.00005, *p* < 0.001]; therefore, degrees of freedom were corrected using Greenhouse-Geisser estimates of sphericity (𝜀 = 0.43). Further analysis within ROIs revealed a significant interaction of valence and origin in **LP** [*F*(4,104) = 4.304, *p* < 0.01]. The *post hoc* tests revealed that amplitude in Null-Pos (*M* = 3.18, *SEM* = 0.34) was significantly more positive than in the Null-Neu [*M* = 2.14, *SEM* = 0.37; *t*(26) = 5.53, *p* < 0.001] condition.

#### Time Window From 350 to 500 ms

A statistically significant interaction between valence and ROIs, *F*(2.91,75.87) = 4.597, *p* < 0.01, was observed. Mauchly’s test revealed that the assumption of sphericity had been violated [χ^2^(6) = 0.02, *p* < 0.001], therefore degrees of freedom were corrected using Greenhouse-Geisser estimates of sphericity (𝜀 = 0.49). Further analysis within ROIs revealed a significant effect of valence in **LP**, *F*(2,52) = 7.372, *p* < 0.01, where the *post hoc* tests showed that the amplitude in Pos (*M* = 1.14, *SEM* = 0.39) is more positive than in Neu [*M* = 0.83, *SEM* = 0.41; *t*(26) = 2.37, *p* = 0.05] and Neg [*M* = 0.53, *SEM* = 0.39; *t*(26) = 4.0, *p* = 0.001]. There was also significant effect of valence in **RP**, *F*(2,52) = 5.9137, *p* < 0.01. The *post hoc* tests showed that amplitude in Neg (*M* = 1.03, *SEM* = 0.51) was less positive than in Neu [*M* = 1.48, *SEM* = 0.52; *t*(26) = 2.42, *p* < 0.05] and Pos [*M* = 1.56, *SEM* = 0.52; *t*(26) = 3.49, *p* < 0.01].

**FIGURE 7:**
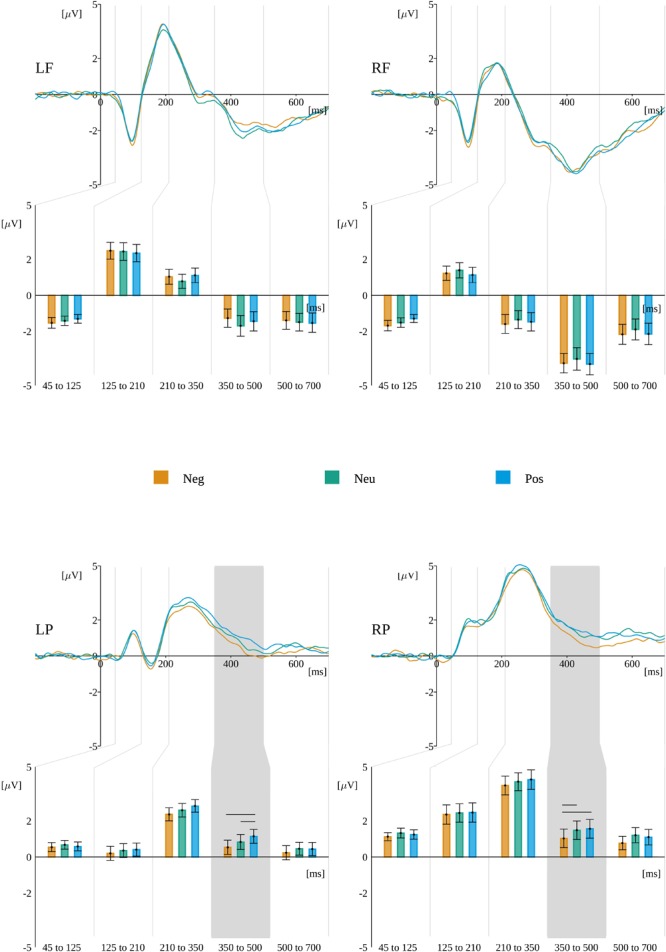
Effects of valence in subsequent time windows during word reading in different ROIs. For each ROI the time course of ERP for each level of valence is plotted. Gray backgrounds mark time windows with statistically significant valence–ROI interactions. Beneath, the bars present the amplitude averaged within each time window. Horizontal lines indicate levels of valence that differ significantly.

**FIGURE 8:**
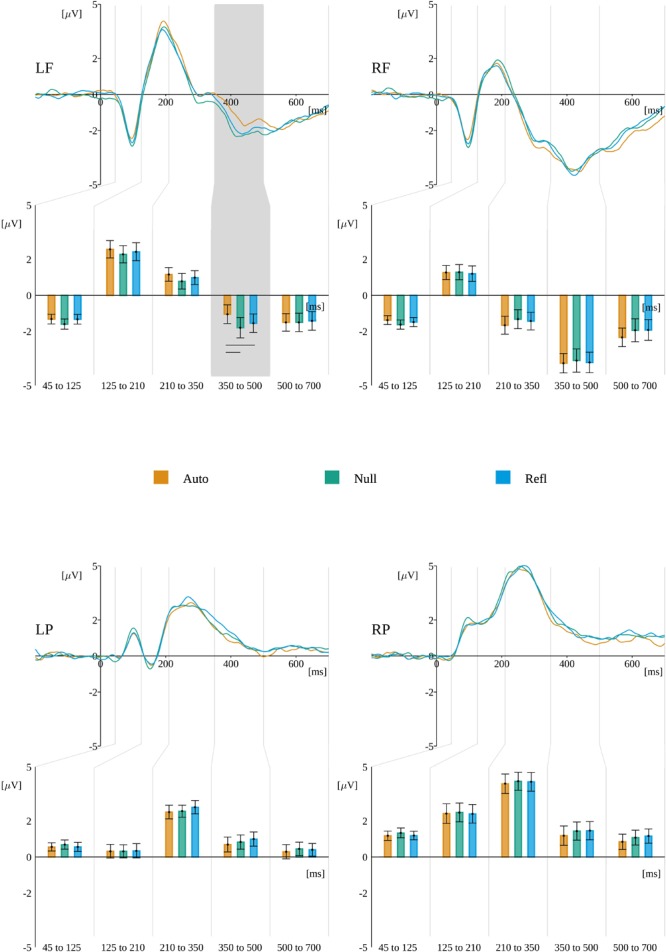
Effects of origin in subsequent time windows during the word reading. For each ROI the time course of ERP for each level of origin is plotted. Gray backgrounds mark time windows with statistically significant origin^∗^ROI interaction. Beneath, the bars present the amplitude averaged within each time window. Horizontal lines indicate levels of origin that differ significantly from each other.

In this time window, a statistically significant interaction between origin and ROIs, *F*(3.34,87.05) = 5.154, *p* < 0.01, was also obtained. Mauchly’s test revealed that the assumption of sphericity had been violated [χ^2^(6) = 0.047, *p* < 0.001]; therefore, degrees of freedom were corrected using Greenhouse–Geisser estimates of sphericity (𝜀 = 0.56). Further analysis within ROIs revealed a significant effect of origin in **LF**, *F*(2,52) = 5.363, *p* < 0.01, where the *post hoc* tests showed that the amplitude in Auto (*M* = -1.05, *SEM* = 0.52) was less negative than in Null [*M* = -1.80, *SEM* = 0.57; *t*(26) = -2.88; *p* < 0.05] and in Refl [*M* = -1.55, *SEM* = 0.52; *t*(26) = -2.89, *p* < 0.05]. The ERP time course and reported effects are presented in Figures 7, 8.

#### Time Window From 500 to 700 ms

There were no statistically significant effects for this time window.

### Decision-Related ERP

#### Time Window From -480 to -330 ms

**FIGURE 9:**
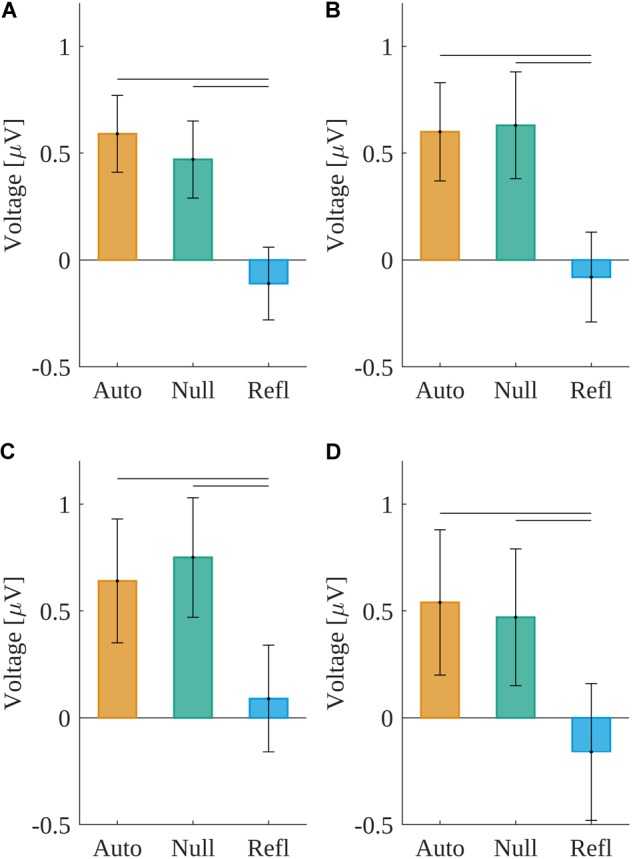
Grand mean amplitudes across participants for origin levels within the time windows: **(A)** from –480 to –330 ms, **(B)** from –330 to –230 ms, **(C)** from –230 to –140 ms, and **(D)** from –140 to 0 ms. Horizontal lines indicate levels of origin that differ significantly from each other.

The main effect of origin, *F*(2,60) = 8.536, *p* ≤ 0.001, was found statistically significant. The amplitude was negative in the Refl condition (*M* = -0.11, *SEM* = 0.17) as opposed to the Auto [*M* = 0.59, *SEM* = 0.18; *t*(60) = -3.48, *p* < 0.005] and Null [*M* = 0.47, *SEM* = 0.18; *t*(60) = -3.35, *p* < 0.005] conditions. Results are presented in Figure 9A.

#### Time Window From -330 to -230 ms

The main effect of origin, *F*(2,60) = 7.741, *p* ≤ 0.001, was found statistically significant. The amplitude was negative in the Refl condition (*M* = -0.08, *SEM* = 0.21) as opposed to the Auto [*M* = 0.6, *SEM* = 0.23; *t*(60) = -3.58, *p* < 0.004] and Null [*M* = 0.63, *SEM* = 0.25; *t*(60) = -3.33, *p* < 0.005] conditions. Results are presented in Figure 9B.

#### Time Window From -230 to -140 ms

The main effect of origin, *F*(2,60) = 4.351, *p* ≤ 0.017, was found statistically significant. The amplitude was significantly less positive in the Refl condition (*M* = 0.09, *SEM* = 0.25) than in the Auto [*M* = 0.64, *SEM* = 0.29; *t*(60) = -2.35, *p* < 0.005] and 0 Null [*M* = 0.75, *SEM* = 0.28; *t*(60) = -2.82, *p* < 0.03] conditions. Results are presented in Figure 9C.

#### Time Window From -140 to 0 ms

There also was a main effect of origin, *F*(2,60) = 5.091, *p* ≤ 0.009. The amplitude was negative in the Refl condition (*M* = -0.16, *SEM* = 0.32) as opposed to the Auto [*M* = 0.54, *SEM* = 0.34; *t*(60) = -2.93, *p* < 0.02] and 0 Null [*M* = 0.47, *SEM* = 0.32; *t*(60) = -2.45, *p* < 0.04] conditions. Results are presented in Figure 9D.

We observed a statistically significant effect between origin and ROIs, *F*(3.48,104.38) = 3.6, *p* < 0.012. Mauchly’s test revealed that the assumption of sphericity had been violated [χ^2^(6) = 0.115, *p* < 0.001]; therefore, degrees of freedom were corrected using Greenhouse–Geisser estimates of sphericity (𝜀 = 0.58).

**FIGURE 10:**
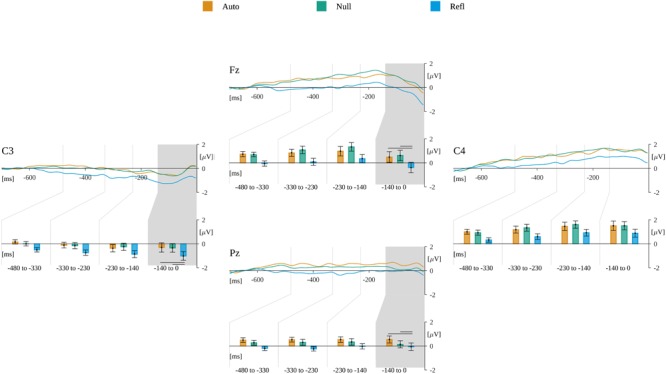
Effects of origin in subsequent time windows during decision-making. For each ROI the time course of ERP for each level of origin is plotted. Gray backgrounds mark time windows with statistically significant origin^∗^ROI interaction. Beneath, the bars present the amplitude averaged within each time window. Horizontal lines indicate levels of origin that differ significantly from each other.

Further analysis within ROIs revealed a significant effect of origin in **Fz**, *F*(2,60) = 5.501, *p* < 0.006, where the *post hoc* tests showed that the amplitude in Refl (*M* = -0.43, *SEM* = 0.44) was more negative than in the Auto [*M* = 0.45, *SEM* = 0.45; *t*(26) = -2.72; *p* < 0.02] and Null [*M* = 0.59, *SEM* = 0.43; *t*(26) = -3.01, *p* < 0.01] conditions. There was also a significant effect found for origin in **C3**, *F*(2,60) = 5.687, *p* < 0.005, where the *post hoc* tests showed that the amplitude in Refl (*M* = -1.01, *SEM* = 0.36) was more negative than in Auto [*M* = -0.29, *SEM* = 0.39; *t*(26) = -2.92; *p* < 0.02] and Null [*M* = -0.33, *SEM* = 0.36; *t*(26) = -2.65, *p* < 0.03]. A significant effect of origin was also found in **Pz**, *F*(2,60) = 3.345, *p* < 0.042, where the *post hoc* tests showed that the amplitude in Refl (*M* = -0.07, *SEM* = 0.31) was more negative than in Auto [*M* = 0.53, *SEM* = 0.3; *t*(26) = -2.71, *p* < 0.03]. A significant effect of origin in **C4**, *F*(2,60) = 4.18, *p* < 0.02, was found; nevertheless, the *post hoc* tests did not show any statistically significant effects. The ERP time course and reported effects are presented in Figure 10.

## Discussion

The aim of the current experiment was to investigate the neural correlates of an interesting behavioral phenomenon, namely, the impact of the origin of affective connotations of word stimuli on the reaction latencies in a task involving intuitive interpretation of ambiguous graphical stimuli. We expected to replicate results of earlier behavioral experiments (Antosz and Imbir, 2017; Imbir, 2017). We also intended to investigate patterns of neural correlates for word valence and origin of affective connotations on both stages of task processing: storing in working memory and decision-making.

### Behavioral Reactions

In the current experiment, reaction latencies in the ambiguous task were determined by two factors: origin and valence of words. The origin effect replicated the main finding of behavioral studies (Antosz and Imbir, 2017; Imbir, 2017): Response latencies were longer for reflective than for automatic originated stimuli and control stimuli (c.f. Figure 2). In the experiment by Imbir (2017), no effect between the control and the reflective-originated condition was found. Considering this, we have to be aware that the nature of words selected for the control condition is such that there is no certain origin associated; therefore, they may be interpreted in terms of automatic or reflective origin by a certain group of participants. Surprisingly, the effect of valence appeared to be significant. This was not the case in earlier studies. Here, negative stimuli correlated with shorter latencies than neutral and positive words. This may be due to the prioritization of processing caused by negative valence, but no ERP results (c.f. Section “The LRP-Like Potential and ERPs During the Decision-Making”) support this claim; therefore, valence effect may be an artifact specific to the current study sample. Alternatively, we may assume this effect to be due to slight differences between the procedures of the current experiment and the previous behavioral ones (Antosz and Imbir, 2017; Imbir, 2017). In the current study, we run the stimuli list three times (c.f. Figure 3), therefore some valence effect associated with habituation could have appeared.

The above discussed effects were not correlated with the type of answer given (QR-code selected from the top or bottom of a screen). The only difference found for this index was that in neutral word conditions, the percentage of “up” responses were lower than in negative and positive conditions. This result do not support the concept of the vertical space metaphor (Lakens, 2012) and its’ linkage to the positivity versus negativity dimension. Earlier studies showed a larger and faster reaction to up-presented positive stimuli and down-presented negative stimuli, analogical to associations of vertical positions with an morality (up)/immorality (down) dimension (Wang et al., 2016), or prioritization of positive valence detections of targets words when they are primed by up arrows than when primed by down arrows, and a reverse pattern observed for negative stimuli (Zhang et al., 2015). We might expect that valence of words should cause a higher percentage of “up” responses for positive stimuli but a lower percentage of “up” responses for negative words. It is worth to highlight, that the current experiment results are not coherent with the space metaphor. On the other hand, in the literature there are data suggesting that the vertical spatial dimension is not directly activated by general valence of words but is activated when a certain word refers to emotional states with stereotypical body postures (Dudschig et al., 2015). Nevertheless, taking into account the main pattern of results, we may assume that replication of behavioral phenomena caused by the origin factor was present (Antosz and Imbir, 2017; Imbir, 2017); thus, neural correlates can be attributed to this phenomena.

### Working Memory Storage During the Word Reading Phase

Considering the first period of processing an ambiguous task, word reading, and storing the word in working memory, we found effects of valence and origin within a 350- to 500-ms time window, which were location-specific. Valence effects were present in posterior ROIs, namely, LP amplitude for positive words was most positive than for other conditions, whereas RP amplitude for negative words was the least positive than for other conditions. It is worth noting that during the whole component amplitudes in posterior ROIs were above zero.

The N400 valence effects typically are caused by incongruence between stimuli (Zhang et al., 2012, 2014; Delaney-Busch and Kuperberg, 2013). In the experiment discussed in this paper, we expected to find differences associated with semantic integration stage of processing, when simpler stimuli (i.e., automatic originated) would evoke less polarized response than more complicated stimuli associated with more complex, multidimensional criteria of evaluation [i.e., reflective originated (Reykowski, 1989; Jarymowicz, 2012; Jarymowicz and Imbir, 2015)]. In other words, the incongruence in current experiment was expected to appear as a result of semantic complexity involving sometimes contradicting one another evaluation criteria leading to reflective reaction to reflective stimuli (Jarymowicz, 2012). For example, the certain object may be evaluated as positive or negative, depends on the viewpoint (like delicious cake evaluated from the short-term pleasure perspective or long-term health and fitness body maintaining perspective). The N400 component was found also to be susceptible to the incongruence in space metaphor. In one study developed to measure event-related potentials (ERPs) in order to explore the nature of space-valence congruency effects, more negative N400 amplitudes were found in the incongruent condition compared with the congruent condition (Zhang et al., 2015), irrespective of stimulus valence. The task for participants was to identify whether the target words had emotional valence; each time a word was primed with up- or down-arrows at the center of the screen in incongruent (e.g., positive and down-arrow) or congruent (e.g., positive and up-arrow) positions with vertical space-metaphor conditions. The valence effects in current experiment were indexed at posterior sites and concerned lower positivity of amplitude for negative words at RP ROI and higher positivity of amplitude for positive words at LP ROI, but general pattern of results was similar at both ROIs (c.f. Figure 7). We may assume, that negative words were less congruent, therefore elicited lower amplitude, but we have to keep in mind the fact that posterior effects were localized at generally positive (above 0 μV) waveform.

The valence results of current experiment were also different that those in the study by Kissler et al. (2007), involving a passive presentation of verbal stimuli differing in the pace of the presentation. The pattern of differences identified there concerned neutral versus emotive stimuli and was present a bit earlier in the EPN component at occipital sites. It is worth noting that in the study by Kissler et al. (2007) recalling was placed at the end of the entire procedure, whereas in the current experiment the time for storing a certain word in working memory was relatively short (2–3 s); but processes for storing and deciding were interfering with one another in the final stage. Therefore, differences in the pattern of results may be understandable. What is more, the stages of discrimination of word valence were identified. Earlier in time effects in amplitude (like in EPN component) differentiated emotive (positive and negative) and neutral words, but later in time effects (like N400 or Late Positive Complex) showed the distinction between amplitudes related to positive or negative words. This is assumed to be connected to processing of meaning (Kissler et al., 2007; Citron, 2012). The current experiment results support this claim (N400 effects for valence).

The origin effects identified in current experiment were localized in left-frontal (LF) ROI. The difference concerned automatic-originated stimuli evoking the least negative amplitude in comparison to other conditions. The frontal localization suggests interpretation of observed differences in terms of an FN400 component (Curran, 2000; Voss and Paller, 2007; Kutas and Federmeier, 2011). The FN400 component is thought to be related to semantic processing, especially the link between stimulus and meaning (Kutas and Federmeier, 2011; Imbir et al., 2016). This pattern appeared to be coherent with our expectations. It is worth comparing the pattern of results for origin to results from an earlier experiment using implicit word processing in the emotional Stroop task (Imbir et al., 2017). The same list of words was used; with no behavioral differences caused by valence and origin, automatic originated words were found to elicit more of a negative amplitude in a 290- to 570-ms time range than reflective originated words. The opposite pattern of differences may account for differences in tasks used. Imbir et al. (2017) depended upon participants’ reaction to each stimulus, whereas the current study involved passive reading, not requiring reactions. Possibly, the automatic origin of the affective meaning of words interferes with decision-making, whereas more complex reflective meaning (Jarymowicz, 2012; Imbir, 2015; Jarymowicz and Imbir, 2015) creates interference (i.e., load) for storing in working memory. In the context of the FN400 component, we may suggest that both control and reflective stimuli required more complex, multidimensional criteria of evaluation [i.e., reflective originated (Reykowski, 1989; Jarymowicz, 2012; Jarymowicz and Imbir, 2015)] than automatic stimuli.

### The LRP-Like Potential and ERPs During the Decision-Making

Taking into account response-locked potentials associated with decision-making, we have obtained the main effect of origin in all analyzed time windows, starting from -480 ms to the decision-execution point (pressing the response key). Each time, the amplitude for decisions concerning reflective originated word stimuli were closer to 0 than amplitudes for decisions in automatic and control conditions. This pattern resembles reaction latencies observed in the current experiment as well as in earlier studies (Antosz and Imbir, 2017; Imbir, 2017). Additionally, an ERP resembling LRP was found for the final time window preceding decision (-140 ms to 0 ms). With the current design of the experiment we cannot demonstrate that it is the same potential as classically described LRP (Coles, 1989; Osman et al., 1995), but the effect of origin was present at the C3 site (contralateral to the responding hand—negative potential), but absent at C4 (positive potential).

It is worth highlighting the fact that a baseline for response-locked potentials was selected within the period starting from -700 and lasting to -600 ms, the interval in which the traces of amplitudes of evoked potentials for different levels of origin were the most similar to one another; therefore, the differences observed for later time ranges (closer to decision) cannot be treated as artifacts. This suggests that although latencies of decision execution differ between conditions, there was a time period close to decision execution when the comparison of physical attributes of stimuli finished and the process of making the final decision began. The effects after -600 ms and before -140 ms can be attributed to the response selection, but the latter potential, similarly as LRP, should represent response execution (Smulders and Miller, 2011). The pattern of results obtained in the current experiment suggest that decisions for reflective-originated stimuli were more difficult not only when planning them; additionally, execution elicited a significantly more negative LRP-like amplitude at the C3 site. The more negative amplitude and longer RTs suggest that participants in reflective-originated conditions had to in fact create a response to a more difficult task (Lin et al., 2017). The difficulty of decision-making may be caused by the process of evidence accumulation (Van Vugt et al., 2014), in the origin case, determined by a richness of semantic relations of multidimensional criteria leading to reflective emotions (Reykowski, 1989; Jarymowicz, 2012; Strack and Deutsch, 2014) in comparison to relatively simple criteria of evaluation characteristics to automatic originated emotions (Damasio, 2010; Jarymowicz, 2012).

### Limitations

The current experiment is the third attempt to explore the behavioral phenomenon based on the origin of an affective state influence on decisions concerning ambiguity. Among the important limitations of the current study, we have to list the procedure of measurement of the LRP-like component, involving responding by only the right hand instead of switching responding hands. Another limitation may be the use of vertically presented stimuli which forces the subject to make saccadic eye movements when changing the fixation point from one QR-code to the other. If the decision process is temporally correlated with the saccadic movement, the applied ICA technique may not separate these two ingredients into separate components. If this is the case removing saccades we removed also the decision related signal. Finally, the effects measured in a response-locked LRP-like component are in fact general and show a low specificity for locations and time frames. This may suggest that the decision making and decision execution stages are distinct one another in time frames and current methodology allowed us to measure the decision execution correlates only, while decision making was not synchronized, therefore no ERPs components related to it were found.

## Conclusion

The pattern of results visualized in the current experiment suggests that in the case of ambiguous task processing (Antosz and Imbir, 2017; Imbir, 2017), we have to deal with distinct processes: reading and storing in working memory a word’s meaning as well as deciding on a certain stimulus. We found origin effects on the FN400 component caused by a complexity of stimuli. Reflective-originated and control stimuli evoked more negative amplitude than automatic-originated. We also found origin-related differences in response-locked potentials, coherent with behavioral differences in reaction latencies. Ambiguous task processing is an interesting paradigm that provides the possibility to investigate the role of affective charge and connotations of the meaning of words for interpretation of unknown stimuli. Ambiguity is a useful testing ground for hypotheses concerning the understanding of how emotions shape our minds when they are at work.

## Author Contributions

All authors contributed to final version of the manuscript. KI: theoretical proposition and results discussion. KI and JŻ: design. KI and MP: method (words). JŻ, JD-G, and GJ: method (EEG measures), experiment execution, and results description. JŻ: experimental procedure programming. JŻ, KI, GJ, and JD-G: statistical analyses. JŻ, KI, JD-G, GJ, and MP: figures.

## Conflict of Interest Statement

The authors declare that the research was conducted in the absence of any commercial or financial relationships that could be construed as a potential conflict of interest. The reviewer FM and handling Editor declared their shared affiliation at the time of the review.

## Footnotes

1 http://braintech.pl/svarog/
