# Supplementary material for: The N400/FN400 and Lateralized Readiness Potential Neural Correlates of Valence and Origin of Words’ Affective Connotations in Ambiguous Task Processing
Source: Front Psychol. 2018 Oct 30;9:1981. doi: 10.3389/fpsyg.2018.01981 (PMC6218570; doi:10.3389/fpsyg.2018.01981)
Supplement: Supplementary file 1 [file Table_1.DOCX]

Appendix

Figure A1. The time course of the ERP for levels of valence preceding the key-press (at time 0) averaged across subjects without baseline correction. Gray rectangles highlight the time range selected for baseline corrections in subsequent analysis.


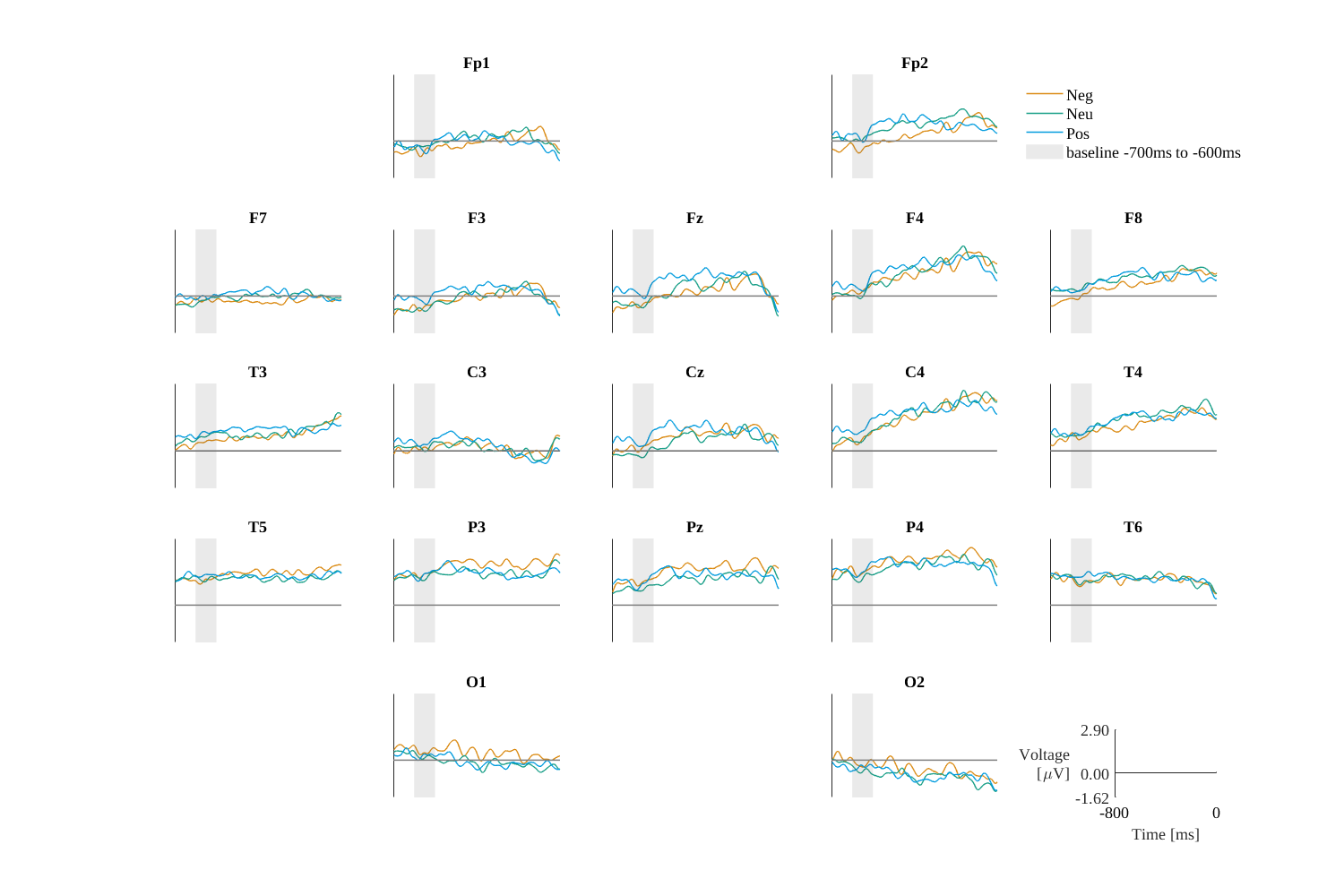


Figure A2. The time course of the ERP for levels of origin preceding the key-press (at time 0) averaged across subjects without baseline correction. Gray rectangles highlight the time range selected for baseline corrections in subsequent analysis.


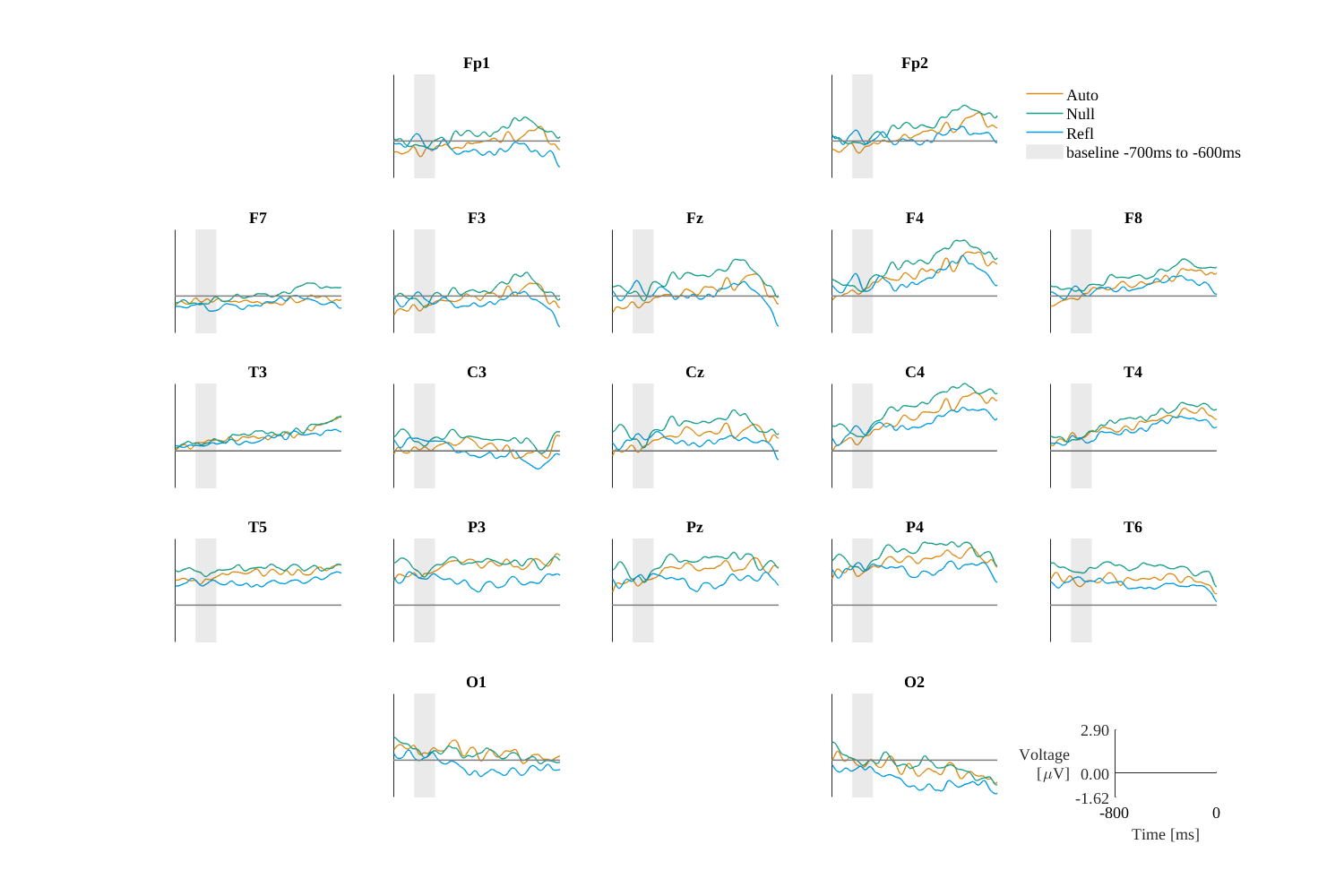


Table A1.

Full list of stimuli with their affective values taken from ANPW_R (Imbir 2016)

| Polish word | English translation | Category number | Category name | valence level | origin level | Valence M | Origin M | Arousal M | Concreteness M | Freqeuncy of appearance | NoL |
| --- | --- | --- | --- | --- | --- | --- | --- | --- | --- | --- | --- |
| czkawka | hiccup | 1 | ANeg | 1 | 1 | 4,04 | 4,54 | 3,86 | 3,18 | 79 | 7 |
| szloch | sob | 1 | ANeg | 1 | 1 | 3,04 | 3,58 | 4,70 | 3,68 | 735 | 6 |
| łzy | tears | 1 | ANeg | 1 | 1 | 3,66 | 3,38 | 4,54 | 2,08 | 660 | 3 |
| uszczypnięcie | pinch | 1 | ANeg | 1 | 1 | 4,06 | 4,38 | 4,38 | 3,44 | 24 | 13 |
| pijak | drunk | 1 | ANeg | 1 | 1 | 2,76 | 4,98 | 5,40 | 2,84 | 467 | 5 |
| naiwniak | sucker | 1 | ANeg | 1 | 1 | 3,38 | 4,16 | 3,84 | 5,40 | 23 | 8 |
| słabeusz | weakling | 1 | ANeg | 1 | 1 | 3,18 | 4,98 | 3,58 | 5,39 | 32 | 8 |
| zmęczenie | fatigue | 1 | ANeg | 1 | 1 | 3,28 | 4,90 | 3,50 | 5,56 | 2044 | 9 |
| hałas | noise | 1 | ANeg | 1 | 1 | 3,56 | 4,74 | 4,60 | 4,18 | 3199 | 5 |
| plotka | rumor | 1 | ANeg | 1 | 1 | 3,50 | 4,78 | 4,48 | 5,04 | 588 | 6 |
| grymas | grimace | 1 | ANeg | 1 | 1 | 3,76 | 4,42 | 4,52 | 4,20 | 1618 | 6 |
| gafa | blunder | 1 | ANeg | 1 | 1 | 3,66 | 4,42 | 4,58 | 5,18 | 23 | 4 |
| usidlenie | ensnaring | 1 | ANeg | 1 | 1 | 3,88 | 4,90 | 4,52 | 5,38 | 9 | 9 |
| smarkacz | stripling | 1 | ANeg | 1 | 1 | 3,36 | 4,88 | 4,64 | 3,46 | 265 | 8 |
| zaślepienie | infatuation | 1 | ANeg | 1 | 1 | 3,32 | 3,66 | 4,40 | 5,64 | 75 | 11 |
| procesja | procession | 2 | ANeu | 2 | 1 | 4,76 | 4,88 | 3,50 | 3,56 | 293 | 8 |
| kościół | church | 2 | ANeu | 2 | 1 | 5,24 | 4,46 | 3,54 | 3,78 | 3652 | 7 |
| kuksaniec | nudge | 2 | ANeu | 2 | 1 | 4,53 | 4,55 | 4,10 | 3,02 | 13 | 9 |
| tarot | tarot | 2 | ANeu | 2 | 1 | 4,16 | 4,90 | 3,72 | 3,92 | 38 | 5 |
| loteria | lottery | 2 | ANeu | 2 | 1 | 5,76 | 4,70 | 4,10 | 3,46 | 56 | 7 |
| westchnienie | sigh | 2 | ANeu | 2 | 1 | 5,48 | 4,28 | 3,60 | 4,44 | 1336 | 12 |
| jałmużna | alms | 2 | ANeu | 2 | 1 | 4,36 | 4,84 | 4,04 | 3,92 | 44 | 8 |
| błazen | clown | 2 | ANeu | 2 | 1 | 4,64 | 4,62 | 4,28 | 3,26 | 507 | 6 |
| mrowienie | tingling | 2 | ANeu | 2 | 1 | 4,26 | 4,90 | 3,96 | 4,39 | 482 | 9 |
| pragnienie | desire | 2 | ANeu | 2 | 1 | 5,14 | 3,40 | 5,18 | 5,80 | 4066 | 10 |
| obrzęd | rite | 2 | ANeu | 2 | 1 | 5,04 | 4,76 | 3,66 | 4,90 | 220 | 6 |
| wróżka | fairy | 2 | ANeu | 2 | 1 | 5,60 | 4,68 | 4,18 | 4,30 | 338 | 6 |
| młodzież | youth | 2 | ANeu | 2 | 1 | 5,68 | 4,50 | 4,66 | 3,40 | 1703 | 8 |
| łasuch | gourmand | 2 | ANeu | 2 | 1 | 5,74 | 4,72 | 4,40 | 4,00 | 9 | 6 |
| burza | storm | 2 | ANeu | 2 | 1 | 4,86 | 4,54 | 5,30 | 3,06 | 3238 | 5 |
| zakochanie | infatuation | 3 | APos | 3 | 1 | 7,56 | 2,28 | 6,50 | 7,24 | 52 | 10 |
| passa | streak | 3 | APos | 3 | 1 | 6,16 | 4,84 | 4,36 | 5,78 | 41 | 5 |
| toast | toast | 3 | APos | 3 | 1 | 6,36 | 4,60 | 4,30 | 3,92 | 689 | 5 |
| powitanie | welcome | 3 | APos | 3 | 1 | 6,50 | 4,96 | 3,90 | 4,70 | 1825 | 9 |
| zapach | fragrance | 3 | APos | 3 | 1 | 6,80 | 4,66 | 3,70 | 4,28 | 9963 | 6 |
| słodycz | sweetness | 3 | APos | 3 | 1 | 7,02 | 4,44 | 4,14 | 3,54 | 477 | 7 |
| pomoc | help | 3 | APos | 3 | 1 | 6,84 | 4,48 | 3,54 | 5,06 | 10180 | 5 |
| niemowlak | infant | 3 | APos | 3 | 1 | 6,50 | 3,86 | 3,70 | 2,50 | 28 | 9 |
| flirt | flirt | 3 | APos | 3 | 1 | 6,46 | 3,36 | 5,52 | 5,72 | 146 | 5 |
| potomstwo | offspring | 3 | APos | 3 | 1 | 6,62 | 4,60 | 3,68 | 3,36 | 504 | 9 |
| pozdrowienie | greeting | 3 | APos | 3 | 1 | 6,72 | 4,60 | 3,58 | 5,40 | 364 | 12 |
| skarb | treasure | 3 | APos | 3 | 1 | 6,84 | 4,76 | 4,20 | 3,72 | 2460 | 5 |
| walentynka | valentine | 3 | APos | 3 | 1 | 6,42 | 4,26 | 4,66 | 4,24 | 2 | 10 |
| podarunek | gift | 3 | APos | 3 | 1 | 6,78 | 4,44 | 4,30 | 3,56 | 373 | 9 |
| ferie | holiday | 3 | APos | 3 | 1 | 7,10 | 4,82 | 4,12 | 4,14 | 123 | 5 |
| wina | fault | 4 | ONeg | 1 | 2 | 3,46 | 5,18 | 4,52 | 5,78 | 9887 | 4 |
| ciemnota | unacquaintance | 4 | ONeg | 1 | 2 | 3,16 | 5,38 | 4,34 | 5,41 | 87 | 8 |
| truchło | carcass | 4 | ONeg | 1 | 2 | 3,56 | 5,16 | 3,98 | 2,90 | 68 | 7 |
| dół | pit | 4 | ONeg | 1 | 2 | 4,04 | 5,52 | 4,02 | 3,28 | 24000 | 3 |
| ochłap | offal | 4 | ONeg | 1 | 2 | 3,28 | 5,60 | 3,80 | 3,72 | 118 | 6 |
| breja | slush | 4 | ONeg | 1 | 2 | 3,49 | 5,90 | 4,10 | 3,11 | 43 | 5 |
| paszkwil | libel | 4 | ONeg | 1 | 2 | 3,57 | 5,48 | 4,51 | 5,38 | 69 | 8 |
| kuternoga | lame | 4 | ONeg | 1 | 2 | 3,77 | 5,07 | 4,13 | 2,96 | 37 | 9 |
| reumatyzm | rheumatism | 4 | ONeg | 1 | 2 | 2,96 | 5,90 | 3,72 | 3,96 | 211 | 9 |
| biedak | wretch | 4 | ONeg | 1 | 2 | 3,48 | 5,18 | 4,16 | 3,22 | 794 | 6 |
| śpiączka | coma | 4 | ONeg | 1 | 2 | 2,44 | 5,36 | 4,02 | 3,70 | 53 | 8 |
| obtarcie | sore | 4 | ONeg | 1 | 2 | 3,46 | 5,06 | 4,22 | 3,20 | 7 | 8 |
| błąd | error | 4 | ONeg | 1 | 2 | 3,38 | 5,46 | 4,34 | 5,68 | 5631 | 4 |
| łachmany | rags | 4 | ONeg | 1 | 2 | 3,26 | 4,98 | 4,30 | 3,04 | 387 | 8 |
| wada | drawback | 4 | ONeg | 1 | 2 | 3,30 | 5,86 | 4,12 | 5,36 | 211 | 4 |
| doping | cheering | 5 | ONeu | 2 | 2 | 5,24 | 5,12 | 5,18 | 4,46 | 23 | 6 |
| chór | choir | 5 | ONeu | 2 | 2 | 5,71 | 5,40 | 3,54 | 2,78 | 1267 | 4 |
| kłębek | hank | 5 | ONeu | 2 | 2 | 5,40 | 5,72 | 3,58 | 2,68 | 1093 | 6 |
| telewizja | television | 5 | ONeu | 2 | 2 | 5,24 | 5,74 | 3,54 | 3,14 | 529 | 9 |
| guru | guru | 5 | ONeu | 2 | 2 | 4,94 | 6,04 | 4,00 | 5,56 | 199 | 4 |
| wódka | vodka | 5 | ONeu | 2 | 2 | 5,60 | 5,10 | 5,76 | 1,58 | 568 | 5 |
| unik | dodge | 5 | ONeu | 2 | 2 | 4,96 | 5,48 | 4,42 | 4,38 | 461 | 4 |
| czara | goblet | 5 | ONeu | 2 | 2 | 5,48 | 5,43 | 3,68 | 2,65 | 214 | 5 |
| smok | dragon | 5 | ONeu | 2 | 2 | 5,66 | 5,14 | 4,58 | 4,18 | 3438 | 4 |
| blef | bluff | 5 | ONeu | 2 | 2 | 4,10 | 5,52 | 4,28 | 5,64 | 136 | 4 |
| żargon | jargon | 5 | ONeu | 2 | 2 | 4,94 | 5,65 | 3,67 | 5,02 | 174 | 6 |
| głębia | depth | 5 | ONeu | 2 | 2 | 5,26 | 5,38 | 3,74 | 5,48 | 243 | 6 |
| farsa | farce | 5 | ONeu | 2 | 2 | 3,98 | 5,08 | 4,54 | 5,54 | 144 | 5 |
| grono | bunch | 5 | ONeu | 2 | 2 | 5,60 | 5,70 | 3,60 | 3,30 | 574 | 5 |
| pisarz | writer | 5 | ONeu | 2 | 2 | 5,74 | 5,92 | 3,76 | 2,98 | 2412 | 6 |
| przyjęcie | party | 6 | OPos | 3 | 2 | 6,78 | 4,96 | 4,54 | 3,88 | 3452 | 9 |
| rejs | cruise | 6 | OPos | 3 | 2 | 6,44 | 5,40 | 3,64 | 3,06 | 689 | 4 |
| powiew | waft | 6 | OPos | 3 | 2 | 6,10 | 5,45 | 3,50 | 3,66 | 1391 | 6 |
| promocja | promotion | 6 | OPos | 3 | 2 | 6,70 | 5,68 | 4,56 | 4,68 | 61 | 8 |
| klimat | climate | 6 | OPos | 3 | 2 | 6,06 | 5,86 | 3,86 | 3,90 | 824 | 6 |
| gość | guest | 6 | OPos | 3 | 2 | 6,42 | 5,38 | 3,76 | 3,28 | 4380 | 4 |
| brawa | applause | 6 | OPos | 3 | 2 | 6,68 | 4,76 | 4,80 | 4,40 | 1053 | 5 |
| kreskówka | cartoon | 6 | OPos | 3 | 2 | 6,82 | 5,14 | 3,96 | 3,04 | 2 | 9 |
| melodia | melody | 6 | OPos | 3 | 2 | 6,88 | 5,24 | 3,46 | 4,50 | 750 | 7 |
| wydarzenie | event | 6 | OPos | 3 | 2 | 5,86 | 5,90 | 4,54 | 4,52 | 1417 | 10 |
| smak | taste | 6 | OPos | 3 | 2 | 6,32 | 5,04 | 3,56 | 4,54 | 2946 | 4 |
| południe | south | 6 | OPos | 3 | 2 | 6,04 | 5,10 | 3,58 | 4,24 | 9135 | 8 |
| malarstwo | painting | 6 | OPos | 3 | 2 | 6,12 | 5,24 | 3,58 | 4,38 | 321 | 9 |
| wyzwanie | challenge | 6 | OPos | 3 | 2 | 6,24 | 5,34 | 4,66 | 5,92 | 1521 | 8 |
| obrońca | defender | 6 | OPos | 3 | 2 | 6,30 | 5,88 | 4,64 | 4,49 | 564 | 7 |
| egzaminy | exams | 7 | RNeg | 1 | 3 | 3,60 | 7,02 | 5,60 | 3,84 | 340 | 8 |
| ignorancja | ignorance | 7 | RNeg | 1 | 3 | 2,98 | 6,14 | 4,68 | 6,44 | 140 | 10 |
| krata | grating | 7 | RNeg | 1 | 3 | 3,98 | 6,16 | 3,60 | 1,74 | 357 | 5 |
| minus | minus | 7 | RNeg | 1 | 3 | 3,84 | 6,36 | 3,52 | 4,90 | 554 | 5 |
| szpieg | spy | 7 | RNeg | 1 | 3 | 3,92 | 6,72 | 4,20 | 3,30 | 637 | 6 |
| koszty | costs | 7 | RNeg | 1 | 3 | 3,78 | 6,40 | 4,08 | 3,88 | 1134 | 6 |
| podwładny | subordinate | 7 | RNeg | 1 | 3 | 4,12 | 6,28 | 4,02 | 4,06 | 189 | 9 |
| podatek | tax | 7 | RNeg | 1 | 3 | 3,32 | 6,92 | 4,22 | 3,60 | 228 | 7 |
| alimenty | alimony | 7 | RNeg | 1 | 3 | 3,60 | 6,34 | 4,48 | 3,42 | 82 | 8 |
| odsetki | interest | 7 | RNeg | 1 | 3 | 3,78 | 6,56 | 4,36 | 3,80 | 58 | 7 |
| rząd | government | 7 | RNeg | 1 | 3 | 3,80 | 6,50 | 4,50 | 3,64 | 5596 | 4 |
| przemyt | smuggling | 7 | RNeg | 1 | 3 | 3,70 | 6,68 | 4,60 | 4,00 | 180 | 7 |
| recesja | recession | 7 | RNeg | 1 | 3 | 3,63 | 6,65 | 4,20 | 5,13 | 25 | 7 |
| bezrobocie | unemployment | 7 | RNeg | 1 | 3 | 2,92 | 6,06 | 4,20 | 5,40 | 122 | 10 |
| heretyk | heretic | 7 | RNeg | 1 | 3 | 3,94 | 6,10 | 4,58 | 5,42 | 45 | 7 |
| szlachta | nobility | 8 | RNeu | 2 | 3 | 5,46 | 6,22 | 4,08 | 3,90 | 811 | 8 |
| etykieta | label | 8 | RNeu | 2 | 3 | 4,90 | 6,60 | 3,52 | 3,08 | 126 | 8 |
| sułtan | sultan | 8 | RNeu | 2 | 3 | 5,10 | 6,84 | 3,46 | 3,10 | 397 | 6 |
| zadatki | makings | 8 | RNeu | 2 | 3 | 5,32 | 5,98 | 3,80 | 5,34 | 94 | 7 |
| prawo | right | 8 | RNeu | 2 | 3 | 5,84 | 7,60 | 3,68 | 5,96 | 19169 | 5 |
| prasa | press | 8 | RNeu | 2 | 3 | 5,30 | 6,64 | 3,50 | 2,84 | 1232 | 5 |
| stawka | bid | 8 | RNeu | 2 | 3 | 5,42 | 6,28 | 4,42 | 4,02 | 301 | 6 |
| raport | report | 8 | RNeu | 2 | 3 | 4,88 | 6,98 | 3,56 | 2,84 | 2634 | 6 |
| wojsko | army | 8 | RNeu | 2 | 3 | 4,94 | 6,62 | 4,96 | 2,90 | 2893 | 6 |
| interes | business | 8 | RNeu | 2 | 3 | 5,86 | 7,10 | 4,36 | 4,82 | 3421 | 7 |
| dyscyplina | discipline | 8 | RNeu | 2 | 3 | 5,46 | 6,44 | 3,96 | 5,74 | 384 | 10 |
| wynik | result | 8 | RNeu | 2 | 3 | 5,52 | 6,70 | 4,60 | 4,58 | 1919 | 5 |
| weto | veto | 8 | RNeu | 2 | 3 | 4,41 | 6,62 | 4,02 | 5,57 | 19 | 4 |
| hodowla | breeding | 8 | RNeu | 2 | 3 | 5,56 | 6,12 | 3,62 | 2,82 | 131 | 7 |
| kurs | course | 8 | RNeu | 2 | 3 | 5,48 | 6,66 | 3,48 | 3,82 | 2801 | 4 |
| miliard | billion | 9 | RPos | 3 | 3 | 7,08 | 7,06 | 4,68 | 4,18 | 311 | 7 |
| tolerancja | tolerance | 9 | RPos | 3 | 3 | 6,62 | 5,32 | 3,88 | 7,32 | 139 | 10 |
| mistrz | master | 9 | RPos | 3 | 3 | 7,22 | 6,24 | 4,52 | 4,38 | 4209 | 6 |
| patent | patent | 9 | RPos | 3 | 3 | 5,92 | 7,59 | 3,52 | 4,62 | 221 | 6 |
| dobytek | property | 9 | RPos | 3 | 3 | 6,48 | 6,76 | 3,74 | 3,78 | 552 | 7 |
| absolwent | graduate | 9 | RPos | 3 | 3 | 6,38 | 6,56 | 3,90 | 3,44 | 206 | 9 |
| uczony | scholar | 9 | RPos | 3 | 3 | 6,26 | 7,44 | 3,58 | 5,04 | 1421 | 6 |
| stypendium | scholarship | 9 | RPos | 3 | 3 | 7,06 | 6,62 | 4,12 | 3,16 | 372 | 10 |
| szczyt | peak | 9 | RPos | 3 | 3 | 6,44 | 6,12 | 4,08 | 3,28 | 3533 | 6 |
| równowaga | balance | 9 | RPos | 3 | 3 | 6,08 | 6,32 | 3,56 | 5,38 | 367 | 9 |
| oszczędności | savings | 9 | RPos | 3 | 3 | 6,68 | 6,94 | 3,94 | 4,40 | 737 | 12 |
| płaca | wages | 9 | RPos | 3 | 3 | 6,16 | 6,82 | 4,27 | 3,27 | 63 | 5 |
| satyra | satire | 9 | RPos | 3 | 3 | 6,04 | 6,16 | 4,04 | 5,10 | 171 | 6 |
| lider | leader | 9 | RPos | 3 | 3 | 6,22 | 6,58 | 4,50 | 4,06 | 90 | 5 |
| zysk | profit | 9 | RPos | 3 | 3 | 6,78 | 6,88 | 4,18 | 4,76 | 651 | 4 |
